# Establishment of a pulmonary epithelial barrier on biodegradable poly-L-lactic-acid membranes

**DOI:** 10.1371/journal.pone.0210830

**Published:** 2019-01-17

**Authors:** Salvatore Montesanto, Natalie P. Smithers, Fabio Bucchieri, Valerio Brucato, Vincenzo La Carrubba, Donna E. Davies, Franco Conforti

**Affiliations:** 1 The Brooke Laboratory, Clinical and Experimental Sciences, Faculty of Medicine, University of Southampton, Southampton, United Kingdom; 2 Department of Civil, Environmental, Aerospace, Materials Engineering (DICAM), University of Palermo, Palermo, Italy; 3 Department of Experimental Biomedicine and Clinical Neurosciences (BIONEC), University of Palermo, Palermo, Italy; 4 Interuniversitary Consortium for Material Science and Technology (INSTM) – Research Unit of Palermo, Palermo, Italy; 5 NIHR Respiratory Biomedical Research Unit, University Hospital Southampton, Southampton, United Kingdom; Hungarian Academy of Sciences, HUNGARY

## Abstract

Development of biocompatible and functional scaffolds for tissue engineering is a major challenge, especially for development of polarised epithelia that are critical structures in tissue homeostasis. Different *in vitro* models of the lung epithelial barrier have been characterized using non-degradable polyethylene terephthalate membranes which limits their uses for tissue engineering. Although poly-L-lactic acid (PLLA) membranes are biodegradable, those prepared via conventional Diffusion Induced Phase Separation (DIPS) lack open-porous geometry and show limited permeability compromising their use for epithelial barrier studies. Here we used PLLA membranes prepared via a modification of the standard DIPS protocol to control the membrane surface morphology and permeability. These were bonded to cell culture inserts for use in barrier function studies. Pulmonary epithelial cells (H441) readily attached to the PLLA membranes and formed a confluent cell layer within two days. This was accompanied by a significant increase in trans-epithelial electrical resistance and correlated with the formation of tight junctions and vectorial cytokine secretion in response to TNFα. Our data suggest that a structurally polarized and functional epithelial barrier can be established on PLLA membranes produced via a non-standard DIPS protocol. Therefore, PLLA membranes have potential utility in lung tissue engineering applications requiring bio-absorbable membranes.

## Introduction

The epithelial barrier of the skin, gastrointestinal and respiratory tract are the main interfaces between our body and the outside environment. Their function is to protect the body from environmental agents including pathogens and pollutants, dehydration, and heat loss. Moreover, the epithelial barriers are essential for the physiological functioning of tissues and organs permitting the formation and maintenance of tissue sub-compartments with different composition. Establishment of specialized cell adhesion complexes, especially tight junctions, are crucial to epithelial barrier integrity and function [1–3]. Conversely, the disruption of tight junction structure, due to specific mutations or altered regulatory signals can result in the development of a range of different diseases [4]. For example, in the lung, disruption of epithelial barrier function has been linked to asthma, COPD and cystic fibrosis [3, 5]. In the 2017 report from the Forum of International Respiratory Societies, respiratory diseases were highlighted as being among the principal causes of severe illness and death worldwide and consequently there is a great need for new and more effective treatments. Furthermore, since the lung epithelium offers a non-invasive and efficient route for the delivery of medical compounds, there is a critical need for methods and models in order to enable investigators to test drugs safety and effectiveness [6, 7].

Due to the complex interaction of genetic and environmental factors in the pathogenesis of human lung diseases, animal models have proved costly and relatively ineffective for lung research [6–8]. Although animal systems are still required by some authorities for pharmaceutical testing or toxicity evaluation, there has been a shift in focus from *in vivo* to *ex vivo* and *in vitro* models of the human lung epithelium [6]. *Ex vivo* models including whole lung perfusion, precision-cut lung slices and biopsy culture are useful models for the evaluation of immune and inflammatory responses as they maintain the *in vivo* tissue architecture and cellular composition, however, they are limited because of their short viability. Furthermore, with the possible exception of whole organ perfusion, exposure of excised tissue samples to challenge agents is not limited to an interaction with the epithelial barrier, as occurs *in vivo*. Different *in vitro* models of the lung epithelial barrier have been well characterized [6, 9]. These involve the use of either immortalised lung cell lines or primary cells from lung biopsy and represent better tools for the development of models that mimic normal or diseased epithelia [6]. However, these models tend to use non-biodegradable scaffolds, for example conventional Transwells with polyethylene terephthalate (PET) membranes, limiting their application in tissue engineering and regenerative medicine. For *in vitro* tissue engineering approaches, non-biodegradable scaffolds limit cell-cell interactions and are structurally different from naturally occurring extracellular matrix (ECM); in contrast biodegradable polymers can be replaced by naturally formed ECM overtime providing the cells with a more normal microenviroment. Extending this concept into regenerative medicine, non-biodegradable scaffolds are more likely to induce inflammatory reactions in the body whereas biodegradable constructs can aid tissue engraphment as they are replaced by naturally produced ECM and their degradation products are non-immunogenic. Therefore, there is a need for development of better support materials for epithelial cells that facilitate the establishment of a functional epithelial barrier while providing biodegradable and biocompatible scaffolds [9].

The development of functional and biocompatible scaffolds is a major challenge in biomedical engineering. Poly-L-lactic acid (PLLA) polymers are widely used for this purpose because of their biodegradability, mechanical properties, and most importantly because of their degradation rate, which is comparable to the healing time in a wound healing situation [10–12]. In particular, these polymers have been optimised for biomedical and pharmaceutical applications with particular focus on orthopaedics for tissue growth implants and fracture fixation devices, drug delivery systems, sutures and soft tissue repair [13, 14]. A conventional technique used for preparation of poly-L-lactic acid (PLLA) membrane is Diffusion Induced Phase Separation (DIPS) that has the advantage of generating membranes with specific characteristics (degradability, strength, morphology and thickness)[15]. However PLLA membranes produced using standard DIPS are only semi-porous, having a nonporous external surface reducing trans-membrane permeability which is critical for basolateral nutrient provision and polarised epithelial cell function [16–18]. Therefore, in order to improve the biocompatibility of PLLA membranes for epithelial cells, it is critical to control the surface characteristics of the membrane during the manufacturing steps. In our previous work [19] we developed a protocol for the preparation of PLLA membranes via a modification of the standard Diffusion Induced Phase Separation (DIPS) with sequential immersion into two coagulation baths. The double bath technique together with the regulation of coagulation bath composition and the desiccation conditions allowed the morphology of membrane surface to be controlled resulting in porous biodegradable PLLA membranes that are potentially more suitable for epithelial cell culture. In this work we demonstrated the formation of a functional lung epithelial barrier on PLLA membranes with optimised ultrastructure and surface characteristics. These may ultimately have application in bioengineering and regenerative medicine.

## Materials and methods

### PLLA membranes

PLLA membranes were made using the poly-L-lactic acid polymer RESOMER L 209 S (Boehringer-Ingelheim, Berkshire, UK) with an inherent viscosity of 3 dl/g using a modification of the standard DIPS method, as previously described [20]. In this work, membranes were prepared via sequential immersion into two coagulation baths. The solvents employed were deionized water and 1,4 dioxane (Sigma-Aldrich, Poole UK). The composition of first coagulation bath was 87:13 dioxane:water (wt:wt) while the second coagulation bath was pure water. The soaking times in the first and second coagulation baths were maintained constant at 5 minutes. After the DIPS process, the resultant membranes were dehydrated in an environment with about 70% relative humidity for 24 h. The surface morphology of the membranes was assessed by scanning electron microscopy (Philips SEM Quanta, FEI) after gold coating to make them conductive. PET membranes were removed from commercial cell culture inserts which were then used as supports for the PLLA membranes. PLLA membranes were cut to size and attached to the base of the cell culture insert by bonding with a biocompatible silicone adhesive (SIMTEC silicone parts, Florida, USA) which was applied to the base the cell culture insert to create a cell culture well. When the adhesive had dried, the entire PLLA culture insert was sterilized using ethanol 70%v/v and dried before use.

### Cell culture and stimulation

NCl-H441 (American Type Culture Collection, HTB-174) cells were obtained from LGC Standards (Teddington, UK) and cultured in RPMI-1640 medium supplemented with 10% fetal bovine serum (FBS), 1mM sodium pyruvate, 100 U/mL penicillin, and 100 μg/mL streptomycin (all from Fisher Scientific-UK Ltd, Loughborough, UK) at 37 °C in a humidified air atmosphere containing 5% CO_2_. For establishment of an epithelial barrier, the H441 cells (1.5*10^5^ cells/insert) were cultured on the apical side of cell culture inserts in the presence of dexamethasone (Sigma-Aldrich, Poole, UK) with 1% insulin-transferrin-sodium selenite (ITS) supplement (Roche Diagnostics Limited, West Sussex, UK). The membrane supports of the cell culture inserts (0.33 cm^2^ cell culture area) were either the PLLA polymer or PET (Transwell Clear inserts (pore size 0.4 μm), Corning, VWR, Dublin, Ireland). Responses of H441 cells to the pro-inflammatory cytokine TNFα (10ng/ml; Sigma-Aldrich, Poole, UK) were assessed on day 4 post-seeding by challenging the apical epithelial surface with the cytokine or vehicle control; after 24h the cell-free culture supernatants from the apical and basal compartments were collected and stored for ELISA, while the cells were fixed for immunofluorescence staining.

### Fluorescent labelling of cells

CellVue Jade Cell Labeling Kit (Fisher Scientific-UK Ltd, Loughborough, UK) was used to evaluate cell monolayer formation on the PLLA membranes. H441 cells were cultured on PLLA cell culture inserts for 48h, washed with PBS and cell membranes were stained using CellVue Jade Cell Labeling Kit. Cell nuclei were counterstained with 4',6-diamidino-2-phenylindole dihydrochloride (DAPI) (1:1000 dilution; Merck Millipore, Darmstadt, Germany). Fluorescent images were acquired using a fluorescence microscope Leica DMI 6000B (Leica Microsystem, Milton Keynes, United Kingdom).

### Hematoxylin and eosin (H&E) staining

H441 cells were cultured in PLLA cell culture inserts for 48h, fixed with 4% formaldehyde (Taab Laboratory Equipment Ltd, Reading, UK) and the excised membranes embedded in paraffin (Leica Microsystems (UK) Ltd, Milton Keynes, UK). H&E staining was performed using a Shandon Varistain 24–4 automatic slide stainer (Fisher Scientific-UK Ltd, Loughborough, UK) on 6 μm sections of the cell-covered membranes.

### Bioelectrical measurements

Transepithelial electrical resistance (TER) was measured daily using STX01 electrodes connected to a Millicell ERS-2 volt-ohm meter (Merck Millipore, Darmstadt, Germany). TER readings (ohms) were corrected for the background value obtained using a PLLA or PET (TER = TER_(cell layer)_-TER_(empty insert)_) cell culture insert containing growth medium alone and then adjusted for the area of the insert (ohms*cm^2^). TER was measured daily after culturing H441 cells in the absence or presence of dexamethasone to assess the formation of a functional epithelial barrier by measuring the electrical resistance.

### Immunofluorescence staining

H441 cells were cultured on PLLA and PET cell culture inserts for 5 days and then fixed with 4% paraformaldehyde followed by permeabilization and staining with occludin-conjugated-Alexa Fluor 488 fluorescent antibody (Fisher Scientific-UK Ltd, Loughborough, UK) (1:100) for the detection of tight junctions [21]. Cell nuclei were counterstained with DAPI. Fluorescent images were acquired using a fluorescence microscope Leica DMI 6000B (Leica Microsystem, Milton Keynes, United Kingdom).

### ELISA analysis

Human Interleukin 8 (IL-8) was evaluated in culture media from the apical and basolateral chambers using an IL-8 DuoSet ELISA (R&D, Abingdon, UK) in accord with manufacturer’s instructions. Each sample was evaluated using 2 technical replicates and the mean value used for subsequent analyses.

### Statistics

Results are expressed as means of ± SD. Differences between groups were assessed using an unpaired T-test or 2way ANOVA for multiple comparisons. All data were analyzed using Prism (GraphPad, CA, USA). p<0.05 was accepted as statistically significant. *p<0.05, **p<0.01, ***p<0.001.

## Results

### Evaluation of cellular monolayer formation on PLLA membranes prepared using a modification of the standard DIPS technique

**Fig 1:**
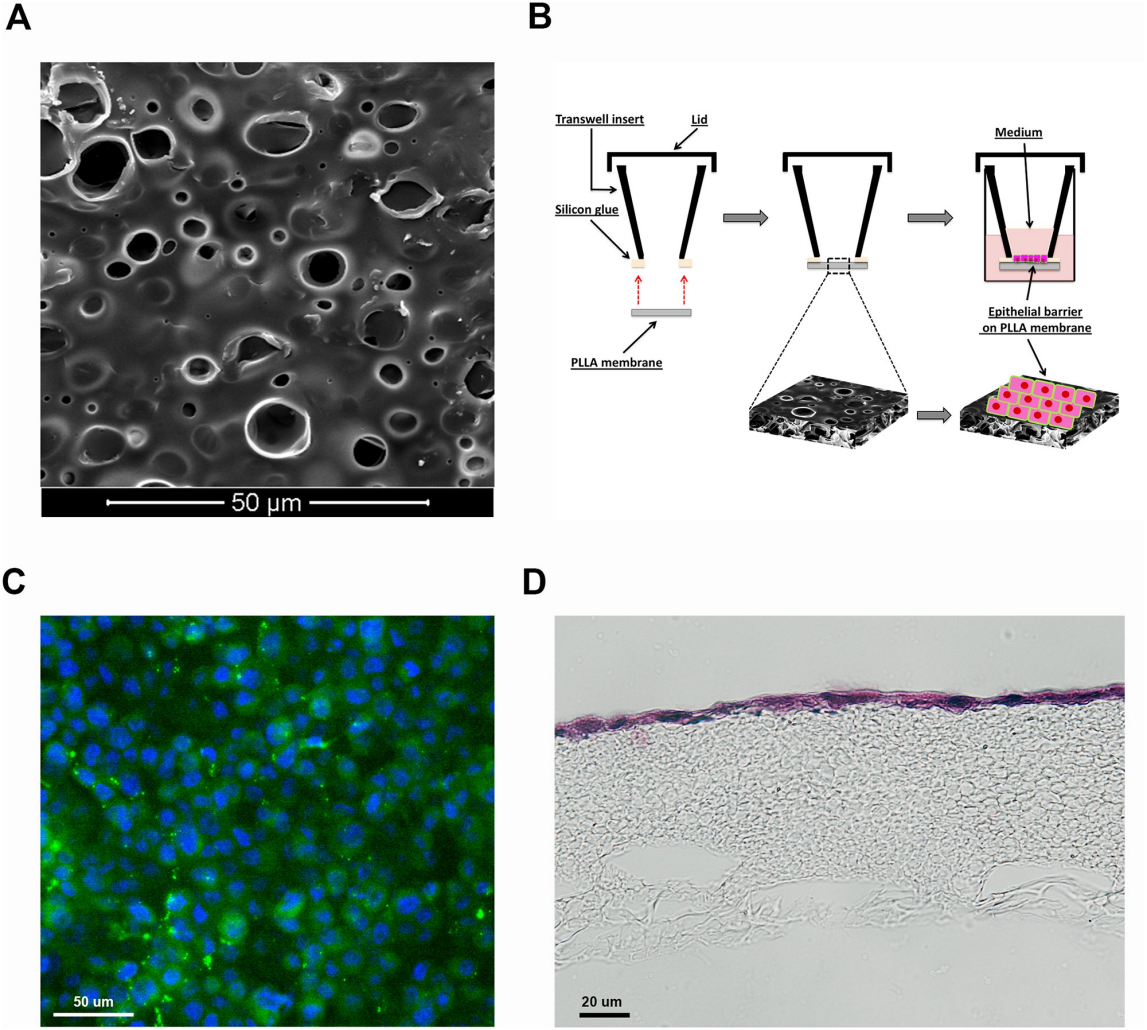
Cell layer formation on PLLA cell culture inserts. A) Morphology of the upper PLLA membrane surface obtained via modification of the standard DIPS method. B) Schematic representation of PLLA cell culture insert. C) An H441 cell monolayer was grown in a PLLA membrane within a cell culture insert and labelled with the fluorescent cell membrane tracker (Blue DAPI and Green Dye-Cell tracker). D) H&E staining of a section of the PLLA membrane covered with an epithelial monolayer after 48 h culture in a cell culture insert.

By using a modification of the standard DIPS method, we produced PLLA membranes with high porosity and permeability characteristics that would be suitable for epithelial barrier studies. Initially, we optimized H441 epithelial cell growth on the PLLA membranes in order to assess the formation of a uniform cell monolayer. The cells were cultured on the upper surface of the PLLA membranes (Fig 1A and 1B) within the cell culture inserts and after 48h they had formed a confluent and homogeneous cell monolayer (Fig 1C and 1D).

### Comparison of PLLA and PET membranes on epithelial paracellular ionic permeability

**Fig 2:**
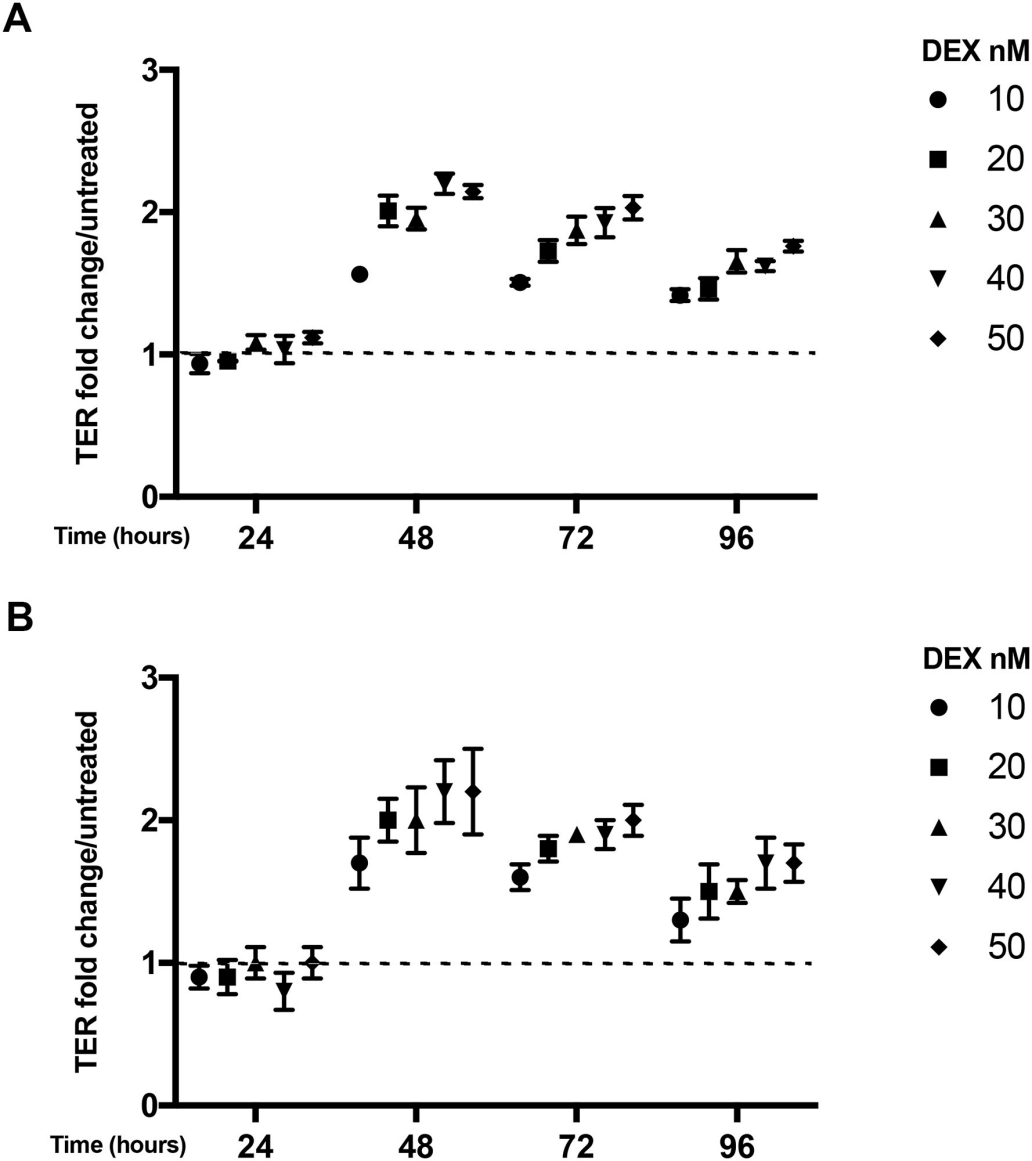
Dexamethasone dose response and time course for H441 barrier formation on PLLA (A) or PET (B) membranes. TER values are expressed as fold change compared to the control (no dexamethasone) on each day (dashed line); maximum TER values were in the range of 359–563 ohms*cm^2^ for PLLA and 455–721 ohms*cm^2^ for PET membranes. TER was calculated as ohms*cm^2^ and corrected for the background value detected in an empty cell culture insert containing medium alone (PET = 54.5 ohms*cm^2^ and PLLA = 99 ohms*cm^2^). Data are mean ± SD, n = 3 independent experiments each performed in duplicate.

Measurement of transepithelial electrical resistance (TER) is used to assess the formation of a functional epithelial barrier on porous membranes where TER reflects the formation of tight junctions and regulation of paracellular ionic permeability across the cell layer [22]. It has previously been shown that H441 cells need dexamethasone for the establishment of a proper epithelial barrier [23]. Therefore, we performed a dose response and time course study of barrier formation in absence and presence of dexamethasone to establish the minimum dose required for maximal polarization while minimising anti-inflammatory effects of the corticosteroid. This showed that after 48h we were able to detect a significant increase in TER in presence of dexamethasone using either PLLA (Fig 2A) or PET (Fig 2B) membranes. Our results showed similar increases in TER for the PLLA membranes compared with PET membranes over both time and doses of dexamethasone tested. Maximal TER values were achieved with Dexamethasone at concentrations > 20 nM. Therefore, we continued our experiments using 20 and 40 nM.

### Formation of tight junctions between epithelial cells cultured on PLLA and PET membranes

**Fig 3:**
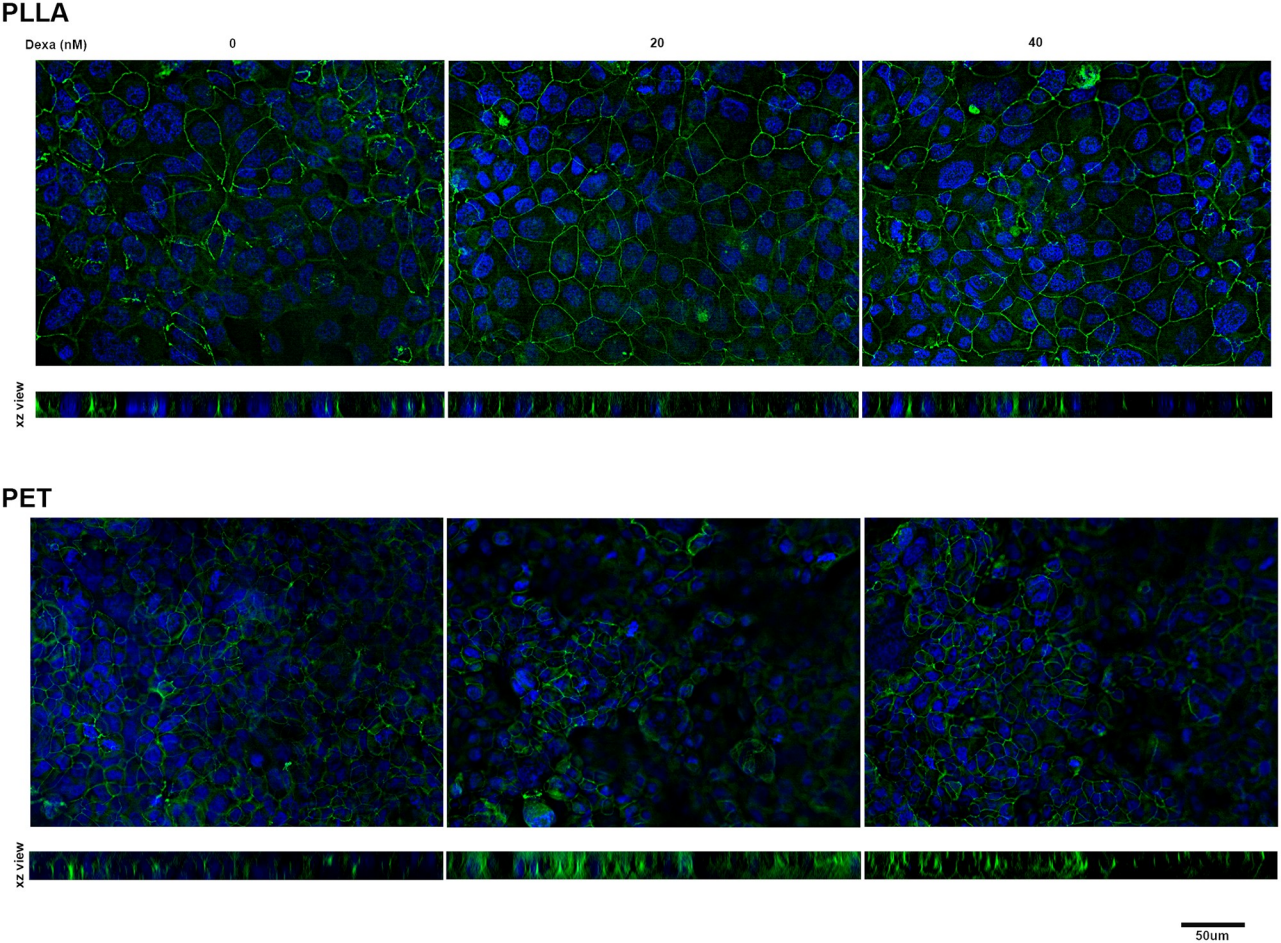
Immunofluorescent images showing tight junctions immunostained using an anti-occludin 488 Alexa Fluor conjugated antibody. Cells were cultured for 5 days on PLLA (upper images) or PET (lower images) membranes without or with dexamethasone treatment (20 and 40 nM). Data are representative of 2 independent experiments.

Cell-cell adhesive interactions and formation of tight junctions is essential for control of paracellular ionic permeability. Therefore, we performed immunofluorescent staining of H441 cells cultured on PLLA or PET membranes to detect occludin, one of the main protein components of tight junctions. For cells cultured in the presence of dexamethasone, the results show clear formation of tight junctions between the cells with a distinct and continuous distribution around the perimeter of each cell (Fig 3), while in absence of dexamethasone the occludin staining was irregular and discontinuous, consistent with the lower TER readings. The formation of tight junctions, as detected by occludin staining, was comparable between PLLA and PET membranes (Fig 3), although it appeared that the cell layer was flatter and more regular on the PLLA membrane perhaps due to their better biomechanical properties.

### Polarised cytokine release in response to the proinflammatory cytokine TNFα

**Fig 4:**
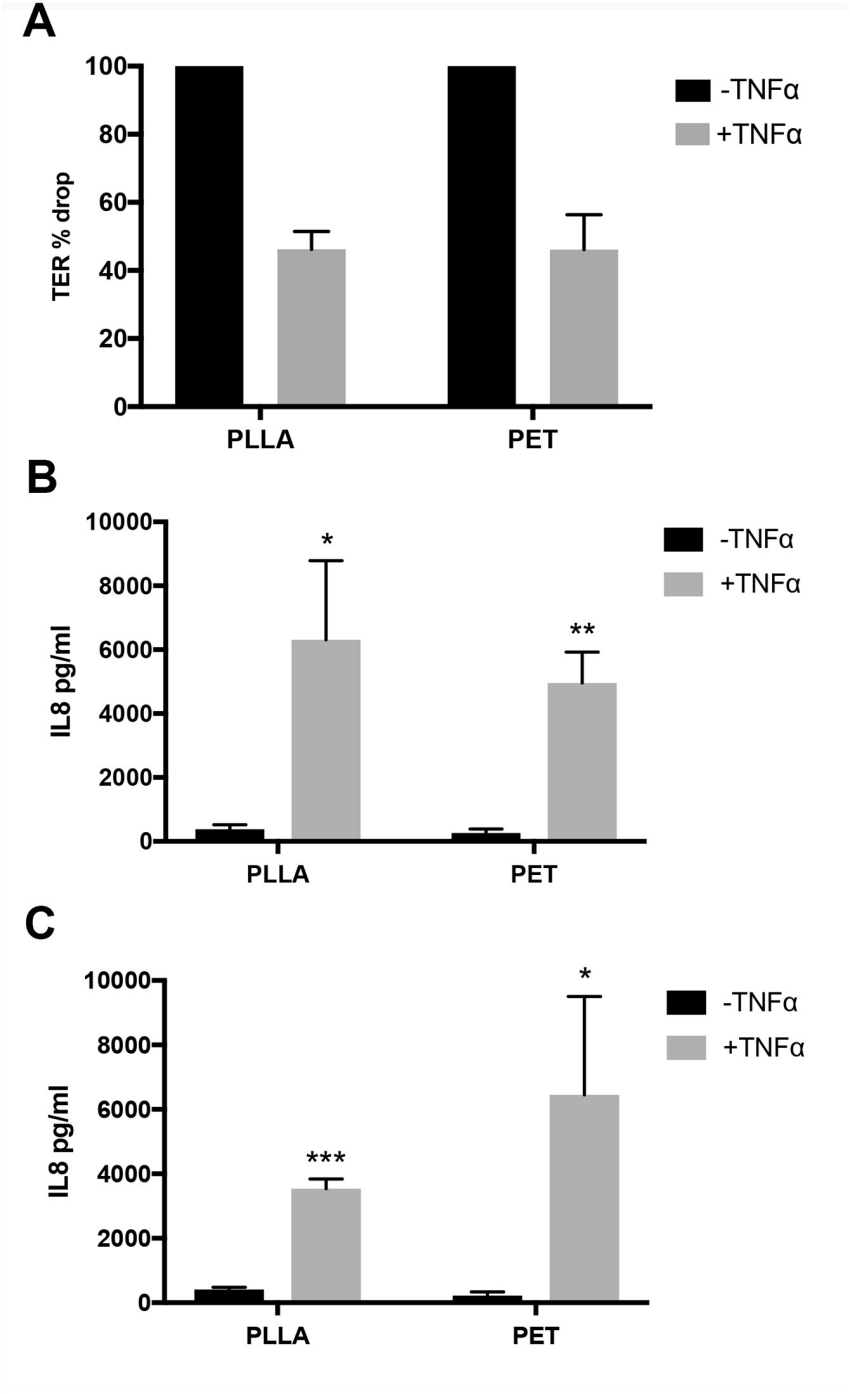
Comparison of the effect of TNFα on polarised epithelial cell layers cultures on PLLA or PET membranes. (A) TER percentage change after 24h treatment with TNFα. (B, C) IL-8 released into apical (B) or basolateral (C) conditioned media of cultures. Data are mean ± SD, n = 3 independent experiments each performed in duplicate.

To further investigate the characteristics of the epithelial monolayer on PLLA membranes, we evaluated the response and functionality of polarised H441 cultures in presence of an inflammatory stimulus. We chose TNFα as it is able to increase epithelial barrier ionic permeability (ie. decrease TER) and induce the release of other inflammatory factors including the chemokine, interleukin 8 (IL-8). Therefore, we established polarised epithelial cell layers on PLLA or PET membranes by 72h of culture with dexamethasone before exposing the apical epithelial surface to TNFα (10ng/ml). This concentration was chosen because it causes the modulation of tight junctions, and can stimulate cytokine release [9]. After 24h of exposure to TNFα, there was a significant decrease in TER compared to untreated controls using H441 cells cultured on either PLLA or PET membranes (Fig 4A) and the increase in epithelial barrier ionic permeability was similar using either type of membrane. Moreover, TNFα was also able to stimulate the release of its downstream mediator [24], IL-8 into both the apical (Fig 4B) and basolateral compartments with no significant difference between cells cultured on either PLLA or PET membranes.

## Discussion and conclusions

Development of biomaterials suitable for both *in vivo* and *in vitro* tissue engineering applications offers opportunities not only for regenerative medicine, but also for target discovery, preclinical evaluation, drug transport and toxicology studies. In our work we show that a functional epithelial barrier can be established on biodegradable PLLA membranes that have enhanced surface porosity and permeability through use of a modification of the standard DIPS protocol. Our results show that the epithelial barrier properties are comparable to those using more conventional PET membranes, however PLLA may be a more suitable material for development of more complex tissue mimetic models because of its biodegradable properties and the possibility of controlling membrane structure. Moreover, PLLA polymers, which are Federal Drug Administration (FDA)-approved, offer an excellent biocompatible and biodegradable scaffold for tissue engraftment *in vivo* where, after its initial role as a cellular support, it can be degraded by physiological processes. This is especially relevant for pulmonary patients with permanent damage, stenosis or a tumour in the trachea; these patients have a poor quality of life because only limited reconstruction options are currently available. However, tissue engineering and regenerative medicine in the lung offers considerable potential [25] and will require establishment of an effective epithelial tissue barrier.

Many techniques have been reported for fabricating porous PLLA membranes. Some of these include methods such as air spinning [26], solvent-cast/particulate leaching [27] and phase separation [16]. Among these techniques, the phase separation approach is one of the most important for obtaining porous structures; such approaches include thermally induced phase separation, air-casting of polymer solution, precipitation from vapour phase, and immersion precipitation [28]. However, DIPS via immersion precipitation is the most widely used membrane preparation method and is an efficient method for the preparation of PLLA scaffolds [16, 29]. Unfortunately, PLLA membranes prepared via conventional DIPS show a non-porous external surface reducing overall membrane permeability and limiting potential applications of PLLA membranes for barrier permeability studies. However, using a modification of the DIPS technique, PLLA membranes can be prepared that allow the morphology of membrane surface to be controlled, resulting in fabrication of porous biodegradable PLLA membranes [19]. Here we show that these membranes can support the growth and polarization of airway epithelial cells. Furthermore, these cultures are functional as shown by regulation of paracellular ionic permeability and vectorial cytokine release in response to TNFα.

The epithelial barrier in the lung is a very dynamic structure that responds to both physiological and pathological stimuli. Its barrier function is achieved by expression of intercellular adhesion complexes, including tight junctions that determine apical-basolateral polarity and control the passage of ions and macromolecules across the lung epithelium [3, 30, 31]. The epithelium also contributes to innate immunity through vectorial release of cytokines and chemokines that signal to cells of the innate and adaptive immune system [32–35]. In our work we successfully established a functional epithelial barrier on a PLLA scaffold prepared using a modified DIPS protocol. We observed the formation of cellular tight junctions resulting in an electrically tight layer of epithelial cells. Moreover, we observed vectorial release of the neutrophil chemokine, IL-8, in response to TNFα. While other studies have tested the utility of PLLA membranes, these have usually been limited to demonstration of their ability to support growth of human cells, including respiratory epithelial cells [36], to date, there has been little functional assessment of biological barrier properties [26, 27, 37]. For example, Selvam et al. used a solvent-cast/particulate leaching technique to fabricate microporous PLLA membranes from PLLA/polyethylene glycol blends and confirmed permeation of glucose, L-tryptophan, and dextran, as well as growth of lacrimal acinar cells which retained histiotypic morphological and physiological characteristics of *in vivo*. However, there was no direct assessment of epithelial barrier formation and function. Similarly, Zhu et al. demonstrated that endothelial cells attach, spread and grow on PLLA membranes modified by immobilization of chitosan, chondroitin sulfate and collagen type I, however they did not study endothelial barrier formation or vectorial responses. This contrasts with our own studies in which we show establishment of tight junctions and an electrically tight ionic barrier. Furthermore, the cellular barrier was able to respond and adapt the physiological stimulus, TNFα, consistent with previous studies using PET membranes [38].

The minimal morphological and structural characteristics of an ideal scaffold for epithelial barrier tissues include offering a support for cell attachment, growth and polarisation, and providing adequate permeability to allow proper exchange of nutrients. However, PLLA scaffolds can offer much more: they can be produced with different morphologies, porosity, surface modifications and degradation times which can be optimised for the cell types under investigation. Furthermore, the microporous nature of PLLA scaffolds can be exploited for infiltration and colonization by mesenchymal cells that synthesise extracellular matrix (ECM) deposition and produce factors that support the barrier tissues. The accumulation of natural ECM while the PLLA membrane gradually dissolves will eventually leave the epithelial cells on a physiologically optimal substrate. Just as epithelial cells provide a barrier to the external environment, endothelial cells form a barrier with the blood compartment, so PLLA scaffolds may also be useful for endothelial barriers studies. In this case, the porosity and biodegradable nature of the scaffold may also facilitate movement of different types of cells that normally circulate in the blood compartment (leukocytes, platelets) into the tissue construct [21, 39, 40]. Thus, ultimately biodegradable PLLA scaffolds may be used to create complex constructs that more closely recapitulate the in vivo tissue environment.

In conclusion, we have used a modification of the standard DIPS protocol to control the membrane surface morphology and permeability of PLLA membranes and demonstrated that they support a functional lung epithelial barrier that dynamically responds to the proinflammatory stimulus, TNFα. Since loss of barrier function has been implicated in respiratory disease like asthma, chronic obstructive pulmonary disease (COPD), acute lung injury (ALI) and fibrosis [2, 4, 30], the results obtained in our work provide new opportunities for the application of PLLA membranes for tissue engineering for target discovery and preclinical testing.

## Supporting information

S1 Table Transepithelial electrical resistance (TER) during time course/dose response experiment. The TER values are the mean of 2 reading in 2 different inserts calculated as ohms*cm^2^ and corrected for the background value detected in an empty cell culture insert containing medium alone (PET = 54.5 ohms*cm^2^ and PLLA = 99 ohms*cm^2^).
(DOCX)

S2 Table IL8 ELISA reading on H441 conditioned media in absence or presence of TNFalpha. (DOCX)

## References

[1] Matthay MA, Ware LB, Zimmerman GA. The acute respiratory distress syndrome. J Clin Invest. 2012;122(8):2731–40. 10.1172/JCI60331 . 22850883 PMC3408735

[2] Davies DE. Epithelial barrier function and immunity in asthma. Ann Am Thorac Soc. 2014;11 Suppl 5:S244–51. 10.1513/AnnalsATS.201407-304AW . 25525727

[3] Loxham M, Davies DE. Phenotypic and genetic aspects of epithelial barrier function in asthmatic patients. J Allergy Clin Immunol. 2017;139(6):1736–51. Epub 2017/06/07. 10.1016/j.jaci.2017.04.005 . 28583446 PMC5457128

[4] Marchiando AM, Graham WV, Turner JR. Epithelial barriers in homeostasis and disease. Annu Rev Pathol. 2010;5:119–44. 10.1146/annurev.pathol.4.110807.092135 . 20078218

[5] Hiemstra PS, McCray PB Jr., Bals R. The innate immune function of airway epithelial cells in inflammatory lung disease. Eur Respir J. 2015;45(4):1150–62. Epub 2015/02/24. 10.1183/09031936.00141514 . 25700381 PMC4719567

[6] Gordon S, Daneshian M, Bouwstra J, Caloni F, Constant S, Davies DE, et al Non-animal models of epithelial barriers (skin, intestine and lung) in research, industrial applications and regulatory toxicology. ALTEX. 2015;32(4):327–78. Epub 2015/11/05. 10.14573/altex.1510051 . 26536291

[7] Vandamme TF. Use of rodents as models of human diseases. J Pharm Bioallied Sci. 2014;6(1):2–9. Epub 2014/01/25. 10.4103/0975-7406.124301 . 24459397 PMC3895289

[8] Holmes AM, Solari R, Holgate ST. Animal models of asthma: value, limitations and opportunities for alternative approaches. Drug Discov Today. 2011;16(15–16):659–70. Epub 2011/07/05. 10.1016/j.drudis.2011.05.014 . 21723955

[9] Schindler M, Nur EKA, Ahmed I, Kamal J, Liu HY, Amor N, et al Living in three dimensions: 3D nanostructured environments for cell culture and regenerative medicine. Cell Biochem Biophys. 2006;45(2):215–27. Epub 2006/06/08. 10.1385/CBB:45:2:215 . 16757822

[10] Cao Y, Vacanti JP, Paige KT, Upton J, Vacanti CA. Transplantation of chondrocytes utilizing a polymer-cell construct to produce tissue-engineered cartilage in the shape of a human ear. Plast Reconstr Surg. 1997;100(2):297–302; discussion 3–4. Epub 1997/08/01. . 9252594 10.1097/00006534-199708000-00001

[11] Ma ZW, Gao CY, Gong YH, Shen JC. Cartilage tissue engineering PLLA scaffold with surface immobilized collagen and basic fibroblast growth factor. Biomaterials. 2005;26(11):1253–9. 10.1016/j.biomaterials.2004.04.031 15475055

[12] Santos AR Jr., Barbanti SH, Duek EA, Dolder H, Wada RS, Wada ML. Vero cell growth and differentiation on poly(L-lactic acid) membranes of different pore diameters. Artif Organs. 2001;25(1):7–13. Epub 2001/02/13. . 11167553 10.1046/j.1525-1594.2001.025001007.x

[13] Saini P, Arora M, Kumar M. Poly(lactic acid) blends in biomedical applications. Adv Drug Deliv Rev. 2016;107:47–59. Epub 2016/07/05. 10.1016/j.addr.2016.06.014 . 27374458

[14] Goncalves F, de Moraes MS, Ferreira LB, Carreira AC, Kossugue PM, Boaro LC, et al Combination of Bioactive Polymeric Membranes and Stem Cells for Periodontal Regeneration: In Vitro and In Vivo Analyses. PLoS One. 2016;11(3):e0152412 Epub 2016/04/01. 10.1371/journal.pone.0152412 . 27031990 PMC4816539

[15] Guillen GR, Pan Y, Li M, Hoek EMV. Preparation and Characterization of Membranes Formed by Nonsolvent Induced Phase Separation: A Review. Industrial & Engineering Chemistry Research. 2011;50(7):3798–817. 10.1021/ie101928r

[16] Montesanto S, Brucato V, La Carrubba V. Evaluation of mechanical and morphologic features of PLLA membranes as supports for perfusion cells culture systems. Mater Sci Eng C Mater Biol Appl. 2016;69:841–9. Epub 2016/09/11. 10.1016/j.msec.2016.07.030 . 27612778

[17] Montesanto S, Fucarino A, Bucchieri F, La Carrubba V, Brucato V. Biological Evaluation of PLLA Membranes, with Different Pore Diameters, to Stimulate Cell Adhesion and Growth in Vitro. Aip Conf Proc. 2015;1695 10.1063/1.4937319

[18] Williams JM, Duckworth CA, Burkitt MD, Watson AJ, Campbell BJ, Pritchard DM. Epithelial cell shedding and barrier function: a matter of life and death at the small intestinal villus tip. Vet Pathol. 2015;52(3):445–55. Epub 2014/11/28. 10.1177/0300985814559404 . 25428410 PMC4441880

[19] Montesanto S, Mannella GA, Carfì Pavia F, La Carrubba V, Brucato V. Coagulation bath composition and desiccation environment as tuning parameters to prepare skinless membranes via diffusion induced phase separation. Journal of Applied Polymer Science. 2015;132(26):n/a–n/a. 10.1002/app.42151

[20] Montesanto S, Bucchieri F, La Carrubba V, and Brucato V. Biological Evaluation of PLLA Membranes, with Different Pore Diameters, to Stimulate Cell Adhesion and Growth in Vitro. American Institute of Physics. (1695, 020041). 10.1063/1.4937319

[21] Xiao C, Puddicombe SM, Field S, Haywood J, Broughton-Head V, Puxeddu I, et al Defective epithelial barrier function in asthma. J Allergy Clin Immunol. 2011;128(3):549–56.e1-12. Epub 2011/07/15. 10.1016/j.jaci.2011.05.038 . 21752437

[22] Lo CM, Keese CR, Giaever I. Cell-substrate contact: another factor may influence transepithelial electrical resistance of cell layers cultured on permeable filters. Exp Cell Res. 1999;250(2):576–80. 10.1006/excr.1999.4538 . 10413610

[23] Salomon JJ, Muchitsch VE, Gausterer JC, Schwagerus E, Huwer H, Daum N, et al The Cell Line NCl-H441 Is a Useful in Vitro Model for Transport Studies of Human Distal Lung Epithelial Barrier. Mol Pharmaceut. 2014;11(3):995–1006. 10.1021/mp4006535 24524365

[24] Kwon OJ, Au BT, Collins PD, Adcock IM, Mak JC, Robbins RR, et al Tumor necrosis factor-induced interleukin-8 expression in cultured human airway epithelial cells. Am J Physiol. 1994;267(4 Pt 1):L398–405. Epub 1994/10/01. 10.1152/ajplung.1994.267.4.L398 . 7943343

[25] Brouwer KM, Hoogenkamp HR, Daamen WF, van Kuppevelt TH. Regenerative medicine for the respiratory system: distant future or tomorrow’s treatment? Am J Respir Crit Care Med. 2013;187(5):468–75. Epub 2012/12/12. 10.1164/rccm.201208-1558PP . 23220914

[26] Francois S, Chakfe N, Durand B, Laroche G. A poly(L-lactic acid) nanofibre mesh scaffold for endothelial cells on vascular prostheses. Acta Biomater. 2009;5(7):2418–28. Epub 2009/04/07. 10.1016/j.actbio.2009.03.013 . 19345622

[27] Selvam S, Chang WV, Nakamura T, Samant DM, Thomas PB, Trousdale MD, et al Microporous poly(L-lactic acid) membranes fabricated by polyethylene glycol solvent-cast/particulate leaching technique. Tissue Eng Part C Methods. 2009;15(3):463–74. Epub 2009/03/06. 10.1089/ten.tec.2008.0431 . 19260769 PMC2821069

[28] Xing Q, Dong X, Li R, Yang H, Han CC, Wang D. Morphology and performance control of PLLA-based porous membranes by phase separation. Polymer. 2013;54(21):5965–73. 10.1016/j.polymer.2013.08.007

[29] Pavia FC, La Carrubba V, Ghersi G, Brucato V. Poly-left-lactic acid tubular scaffolds via diffusion induced phase separation: Control of morphology. Polymer Engineering & Science. 2013;53(2):431–42. 10.1002/pen.23273

[30] Brune K, Frank J, Schwingshackl A, Finigan J, Sidhaye VK. Pulmonary epithelial barrier function: some new players and mechanisms. Am J Physiol Lung Cell Mol Physiol. 2015;308(8):L731–45. Epub 2015/02/01. 10.1152/ajplung.00309.2014 . 25637609 PMC4747906

[31] Higuita-Castro N, Nelson MT, Shukla V, Agudelo-Garcia PA, Zhang W, Duarte-Sanmiguel SM, et al Using a Novel Microfabricated Model of the Alveolar-Capillary Barrier to Investigate the Effect of Matrix Structure on Atelectrauma. Sci Rep. 2017;7(1):11623 Epub 2017/09/16. 10.1038/s41598-017-12044-9 . 28912466 PMC5599538

[32] Blume C, Swindle EJ, Gilles S, Traidl-Hoffmann C, Davies DE. Low molecular weight components of pollen alter bronchial epithelial barrier functions. Tissue Barriers. 2015;3(3):e1062316 Epub 2015/10/10. 10.1080/15476286.2015.1062316 . 26451347 PMC4574901

[33] Chow AW, Liang JF, Wong JS, Fu Y, Tang NL, Ko WH. Polarized secretion of interleukin (IL)-6 and IL-8 by human airway epithelia 16HBE14o- cells in response to cationic polypeptide challenge. PLoS One. 2010;5(8):e12091 Epub 2010/08/17. 10.1371/journal.pone.0012091 . 20711426 PMC2920803

[34] Whitsett JA, Alenghat T. Respiratory epithelial cells orchestrate pulmonary innate immunity. Nat Immunol. 2015;16(1):27–35. Epub 2014/12/19. 10.1038/ni.3045 . 25521682 PMC4318521

[35] Hermanns MI, Kasper J, Dubruel P, Pohl C, Uboldi C, Vermeersch V, et al An impaired alveolar-capillary barrier in vitro: effect of proinflammatory cytokines and consequences on nanocarrier interaction. J R Soc Interface. 2010;7 Suppl 1:S41–54. Epub 2009/10/02. 10.1098/rsif.2009.0288.focus . 19793744 PMC2843988

[36] Ostwald J, Dommerich S, Nischan C, Kramp B. [In vitro culture of cells from respiratory mucosa on foils of collagen, poly-L-lactide (PLLA) and poly-3-hydroxy-butyrate (PHB)]. Laryngorhinootologie. 2003;82(10):693–9. Epub 2003/11/01. 10.1055/s-2003-43238 . 14593567

[37] Zhu Y, Gao C, Liu Y, Shen J. Endothelial cell functions in vitro cultured on poly(L-lactic acid) membranes modified with different methods. J Biomed Mater Res A. 2004;69(3):436–43. Epub 2004/05/06. 10.1002/jbm.a.30007 . 15127390

[38] Hardyman MA, Wilkinson E, Martin E, Jayasekera NP, Blume C, Swindle EJ, et al TNF-alpha-mediated bronchial barrier disruption and regulation by src-family kinase activation. J Allergy Clin Immunol. 2013;132(3):665–75 e8. Epub 2013/05/02. 10.1016/j.jaci.2013.03.005 . 23632299

[39] Granger DN, Senchenkova E. Inflammation and the Microcirculation. Integrated Systems Physiology-From Cell to Function. San Rafael (CA) 2010. 21452440

[40] Granger DN, Kubes P. The microcirculation and inflammation: modulation of leukocyte-endothelial cell adhesion. J Leukoc Biol. 1994;55(5):662–75. Epub 1994/05/01. . 8182345

