# Supplementary material for: Establishment of a pulmonary epithelial barrier on biodegradable poly-L-lactic-acid membranes
Source: PLoS One. 2019 Jan 17;14(1):e0210830. doi: 10.1371/journal.pone.0210830 (PMC6336298; doi:10.1371/journal.pone.0210830)
Supplement: S1 Table — The TER values are the mean of 2 reading in 2 different inserts calculated as ohms*cm2 and corrected for the background value detected in an empty cell culture insert containing medium alone (PET = 54.5 ohms*cm2 and PLLA = 99 ohms*cm2). (DOCX) [file pone.0210830.s001.docx]

**S1 Table. Transepithelial electrical resistance (TER) during time course/dose response experiment.**

The TER values are the mean of 2 reading in 2 different inserts calculated as ohms*cm^2^ and corrected for the background value detected in an empty cell culture insert containing medium alone (PET=54.5 ohms*cm^2^ and PLLA=99 ohms*cm^2^).
