# Supplementary material for: Establishment of a pulmonary epithelial barrier on biodegradable poly-L-lactic-acid membranes
Source: PLoS One. 2019 Jan 17;14(1):e0210830. doi: 10.1371/journal.pone.0210830 (PMC6336298; doi:10.1371/journal.pone.0210830)
Supplement: S2 Table — (DOCX) [file pone.0210830.s002.docx]

**S2 Table. IL8 ELISA reading on H441 conditioned media in absence or presence of TNFalpha.**

| **IL8 ELISA PLLA membrane** |  |  |  |  |  |
| --- | --- | --- | --- | --- | --- |
|  |  | apical | basolateral | apical | basolateral |
|  |  | -TNFalpha | -TNFalpha | +TNFalpha | +TNFalpha |
| **1st Experiment** | pg/ml reading 1 | 509.47 | 459.39 | 4135.56 | 3898.40 |
|  | pg/ml reading 2 | 494.06 | 472.61 | 4234.94 | 3617.07 |
|  |  |  |  |  |  |
| **2nd Experiment** | pg/ml reading 1 | 240.27 | 339.21 | 8052.25 | 3180.53 |
|  | pg/ml reading 2 | 264.24 | 368.01 | 8824.91 | 3468.49 |
|  |  |  |  |  |  |
| **3rd Experiment** | pg/ml reading 1 | 431.09 | 384.56 | 7013.21 | 3176.76 |
|  | pg/ml reading 2 | 388.79 | 369.47 | 6710.59 | 3505.51 |
|  |  |  |  |  |  |
| **IL8 ELISA PET membrane** |  |  |  |  |  |
|  |  | apical | basolateral | apical | basolateral |
|  |  | -TNFalpha | -TNFalpha | +TNFalpha | +TNFalpha |
| **1st Experiment** | pg/ml reading 1 | 317.16 | 207.82 | 5343.46 | 2564.22 |
|  | pg/ml reading 2 | 274.59 | 214.23 | 5383.38 | 2670.96 |
|  |  |  |  |  |  |
| **2nd Experiment** | pg/ml reading 1 | 398.02 | 356.39 | 5692.49 | 8198.33 |
|  | pg/ml reading 2 | 369.43 | 355.84 | 5848.96 | 7788.74 |
|  |  |  |  |  |  |
| **3rd Experiment** | pg/ml reading 1 | 103.79 | 85.16 | 3912.51 | 9732.43 |
|  | pg/ml reading 2 | 109.46 | 87.51 | 3566.06 | 7745.05 |
